# Duckweed as a Sustainable Aquafeed: Effects on Growth, Muscle Composition, Antioxidant and Immune Markers in Grass Carp

**DOI:** 10.3390/ani16010053

**Published:** 2025-12-24

**Authors:** Yingjie Song, Zhangli Hu, Xuewei Yang, Yuxing An, Yinglin Lu

**Affiliations:** 1 Institute of Nanfan and Seed Industry, Guangdong Academy of Sciences, Guangzhou 510220, China; 2 Guangdong Provincial Key Laboratory of Functional Substances in Food and Medicine and Disease Prevention in Eastern Guangdong, Hanshan Normal University, Chaozhou 521000, China; 3 College of Life Sciences and Oceanography, Shenzhen University, Shenzhen 518060, China

**Keywords:** *Spirodela polyrhiza*, aquafeed, bioactive components, nutritional quality, gut microbiota

## Abstract

Duckweed (*Spirodela polyrhiza*), a fast-growing aquatic plant rich in protein and bioactive compounds, offers a sustainable alternative to conventional aquafeed protein sources. This study evaluated the effects of incorporating 25–75% duckweed meal into a commercial feed on grass carp (*Ctenopharyngodon idella*) over a 6-week trial. Fish meal, wheat starch, and vegetable oil was added in amounts to obtain isonitrogenous and isoenergetic diets. Additionally, another grass carps were used for extended feeding until they reached approximately 1000 g, using the feed with the optimal duckweed inclusion rate (25%). Fish fed a diet consisting of 75% commercial feed and 25% duckweed meal (F75D25) exhibited significantly higher weight gain. Muscle analysis revealed increased protein content (up 15%, *p* < 0.05) and improved amino acid and fatty acid profiles. Liver, muscle, and blood assays showed elevated antioxidant enzyme activities (SOD up 20%, LYS up 18%; *p* < 0.05) and immune markers (CRP, GOT; *p* < 0.05), indicating enhanced health status. Transcriptomic and metagenomic analyses confirmed the upregulation of immune-related genes (e.g., SOD1, IL-6; fold change > 2, *p* < 0.01) and beneficial shifts in gut microbiota (e.g., increased Firmicutes). These findings highlight duckweed’s potential as a nutrient-rich, health-promoting ingredient for sustainable aquaculture diets.

## Simple Summary

Feeding fish is one of the biggest costs in aquaculture, and traditional ingredients such as soybean meal and fishmeal are becoming more expensive and place pressure on the environment. Duckweed, a small floating water plant, grows very fast and is rich in protein and healthy compounds. This study tested whether duckweed could constitute part of the normal diet of grass carp, one of the most important farmed fish in Asia. We compared diets where commercial feed was partly or fully incorporated with duckweed. The best results were found when 25% of the feed was incorporated. Fish in this group grew faster, used their food more efficiently, and developed better muscle quality than fish fed only commercial feed. We also observed improvements in fish health, including stronger antioxidant defenses and better immune responses. The gut bacteria of these fish also shifted toward more beneficial types. These results show that duckweed is a promising and environmentally friendly feed ingredient. Using duckweed could lower costs for farmers, reduce reliance on conventional feed ingredients, and improve the sustainability of aquaculture.

## 1. Introduction

*Spirodela polyrhiza*, commonly known as greater duckweed, is a free-floating, rapidly proliferating aquatic monocot. It is ubiquitously found in stagnant and slow-flowing aquatic environments worldwide. *S. polyrhiza* is characterized by its simplicity and lack of specialized structures such as leaves or stems. Instead, it possesses flat, ovoid, leaf-like structures called fronds, accompanied by a single rootlet that aids in stabilization [1]. The fronds exhibit a bright green color on the upper surface and a purple hue on the lower surface, which not only contributes to its aesthetic appeal but also makes it an attractive choice for use in aquariums [2]. *S. polyrhiza* as the most basal member of the Lemnaceae family, the utility and potential of *S. polyrhiza* have gained recognition in various domains. This species is notably effective in wastewater treatment, especially in the removal of nitrogen [3,4]. Additionally, it has been explored for biofuel production [5,6], recombinant protein production [7,8] and carbon sequestration [9,10,11].

In the Chinese Pharmacopoeia, it is recorded that *S. polyrhiza* has the effect of clearing heat and removing toxins. All of these effects have been proven to originate from the bioactive components in *S. polyrhiza*, including flavonoids, polysaccharides, alkaloids, and fatty acids [12,13,14,15]. Above all, *S. polyrhiza* exhibits a broad spectrum of medicinal properties, including antioxidant, anti-inflammatory, antimicrobial, diuretic, hemostatic, dermatological, and ophthalmic benefits [13,14,16]. In *S. polyrhiza* the protein content is notably high, making up approximately 35–45% of its dry weight [17]. This substantial protein concentration highlights its potential as a valuable source of bioactive peptides and enzymes [18]. In the pharmaceutical sector, antioxidant enzymes such as SOD and CAT from *S. polyrhiza* are being explored for their potential in mitigating oxidative stress-related disorders [19]. Additionally, the high protein content supports its use in nutraceuticals, where *S. polyrhiza* can serve as a protein supplement in human and animal diets, offering a sustainable and cost-effective alternative to traditional protein sources [20].

*S. polyrhiza* possesses significant potential as a sustainable feed source in aquaculture and livestock industries due to its high nutritive value, rapid growth rate, and low environmental footprint. Characterized by a high protein content, it also contains essential amino acids [8], vitamins (such as A, B, and C) [21], minerals (including phosphorus, potassium, and magnesium) [22], and fibers [23] making it an excellent inclusion ingredient in animal diets. And *S. polyrhiza* has used supplement for fish, pigs and poultry [24,25,26,27]. *S. polyrhiza* presents a cost-effective alternative to conventional feed ingredients like soybean meal and fishmeal, whose prices are subject to global market fluctuations. The minimal requirement for land and the lower water footprint associated with duckweed cultivation further enhance its attractiveness as a feed source.

Grass carp (*Ctenopharyngodon idella*) is one of the most widely cultured herbivorous freshwater fish species in China and Southeast Asia, known for its rapid growth, high feed conversion efficiency, and strong market demand [28,29]. However, the increasing cost and environmental footprint of conventional protein sources such as fishmeal and soybean meal highlight the need for sustainable alternatives. In this study, we hypothesized that incorporating different proportions of duckweed meal (25–100%) into the commercial feed would influence growth performance, nutrient utilization, immune status, and gut microbial balance in grass carp, and that an optimal inclusion level could be identified. The objective was to evaluate the comprehensive effects of duckweed inclusion on growth performance, flesh quality, physiological health, gene expression, and gut microbial structure in a controlled feeding trial.

## 2. Materials and Methods

### 2.1. Fish and Diets

Grass carp (body weight 7.34 ± 0.32 g, body length 5.12 ± 0.24 cm) were obtained from Guangzhou Tianfa Fish Farm (Huadu, Guangdong Province, Guangzhou, China). All fish were cultivated in glass aquarium (5.0 m × 1.2 m × 1.0 m) with continuous system of water filtration and aeration (photoperiod 16:8 h light and dark cycle) at constant temperature (25.0 ± 1 °C) at Shenzhen university. A total of 300 fish were randomly selected and evenly distributed into five groups to conduct an experimental trial aimed at determining the optimal inclusion rate of duckweed in their feed. Additionally, another 300 grass carp (initial average weight 7.34 ± 0.32 g, body length 5.12 ± 0.24 cm) were used for extended feeding until they reached approximately 1000 g, using the feed with the optimal duckweed inclusion rate. Fish were fed twice daily at 3% body weight, under the same rearing conditions, until reaching 1000 g (approximately 3 months). The feed selected was the fish-specific expanded compound feed produced by Guangdong Tongwei Feed Co., Ltd (Guangzhou, China). The commercial feed (Guangdong Tongwei Feed Co., Ltd., Guangzhou, China) had the following ingredient composition: soybean meal (40%), rapeseed meal (20%), wheat flour (15%), fishmeal (10%), vegetable oil (5%), vitamins/minerals (5%), and other additives (5%). Proximate nutrient composition: crude protein 28%, crude lipid 4.45%, ash 9.56%, starch 30.48% (analyzed per GB5009 standards). Duckweed (*S. polyrhiza*) biomass was harvested from a controlled aquaculture facility supplied with clean freshwater, rather than from polluted or wastewater environments. The duckweed biomass was air-dried under shade for approximately 48 h and subsequently dried in a forced-air drying chamber at 60 °C for 12 h until a constant weight was achieved. The dried duckweed was then ground into a fine powder prior to feed formulation. Although no separate toxicity assays were performed in this study, the source and preparation procedures ensured that the material was safe for use in feed formulation.

Feed Preparation. Five experimental diets were prepared with different inclusion levels of *Spirodela polyrhiza* duckweed meal into a commercial feed matrix: F100D0 (100% commercial feed, CF), F75D25 (75% CF + 25% duckweed meal), F50D50 (50% CF + 50% duckweed meal), F25D75 (25% CF + 75% duckweed meal), and F0D100 (100% duckweed meal). These diet codes represent the nominal inclusion ratios of commercial feed and duckweed in the basal mixture, rather than the replacement of specific feed ingredients.

The nutritional composition of the commercial feed and duckweed meal is presented in Table 1. Based on these data, the experimental diets were formulated on a dry-matter basis using commercial feed and duckweed meal as basal ingredients, while small amounts of fish meal, wheat starch, and vegetable oil were externally added as balancing ingredients to obtain approximately isonitrogenous and isoenergetic diets (Table 2). The quantities of fish meal, wheat starch, and vegetable oil reported in Table 2 therefore represent the actual added amounts (g/kg DM) and do not correspond to intrinsic components of the commercial feed. With increasing duckweed inclusion, the proportion of commercial feed was progressively reduced, whereas the added balancing ingredients were adjusted to minimize differences in dietary protein and energy levels among treatments.

Feed processing and pelleting. Duckweed biomass used in this study was harvested from a controlled freshwater culture system supplied with clean water and not from wastewater or contaminated environments, rinsed with clean water to remove surface impurities, and air-dried under shade for approximately 48 h until reaching a constant weight. The partially dried biomass was further dried in a forced-air drying chamber at 60 °C for 12 h to ensure uniform moisture removal. Dried duckweed was then ground into a fine meal using a laboratory mill and passed through a 0.5-mm mesh sieve prior to feed formulation.

For diet preparation, commercial feed, duckweed meal, and the added balancing ingredients (fish meal, wheat starch, and vegetable oil) were weighed according to the formulations shown in Table 2 and thoroughly mixed in a ribbon mixer. The mixtures were conditioned with steam at approximately 70–75 °C for 20–30 s, and then pelleted using a hand-pelleted to produce pellets with a diameter of 2–3 mm. The freshly pelleted diets were dried in a ventilated oven at 50 °C to constant weight, cooled to room temperature, and stored in sealed bags at 4 °C until use.

Each treatment included three replicate tanks (30 L) with 20 grass carp per tank. Fish were euthanized using MS-222 (200 mg/L) at the end of the 6-week trial or at sampling points. Blood, muscle, gut, and liver tissues were collected and stored at −80 °C for analyses of growth performance, muscle quality, biochemical indicators, transcriptomics, and gut microbiota composition.

### 2.2. Experimental Set-Up

Five different treatments were applied in the experiment (F100D0, F75D25, F50D50, F25D75 and F0D100). This study spanned six weeks, during which the growth rates and feed intake of the fish were monitored to compute the feed conversion ratio (FCR). Measurements of body length were conducted using a centimeter ruler, whereas fish weights were determined with an analytical balance. Both weight and length assessments were performed at the conclusion of the experimental period.

The optimal F75D25 diet was used for extended feeding (control: F100D0). Rearing conditions were the same as in the initial trial. Growth was monitored bi-weekly; endpoint at ~1000 g chosen for market-size analysis. Sampling at 1 month (early effects) and 3 months (long-term) to assess temporal changes. Sampling: 6 fish per group euthanized humanely (MS-222 anesthesia, 200 mg/L), dissected for tissues.

### 2.3. Feed Conversion Ratio

To calculate the FCR over the course of the experimental period, the weight gain of the fish was quantified throughout the 6-week duration of this study:

Weight gain=Final weight−Inital weight

The FCR was calculated using the following formula:

FCR=Feed intake/Weight gain g/g

Feed intake for each treatment group was calculated as the difference between the amount of feed offered and the uneaten feed collected by siphon one hour after feeding. This approach ensured that actual consumption was measured rather than assumed.

### 2.4. Analysis of Muscle Quality, Physiological and Biochemical Indicators of C. idella

The moisture, total protein, lipids, ash, carbohydrate content and compositions of the amino acid and fatty acid of the dorsal muscle of *C. idella* were analyzed. Total protein content was determined by Kjeldahl nitrogen analyzer (K9840, Jinan Hanon Instruments, Jinan, China). The moisture, ash, carbohydrate content were determined using GB5009.3-2016 [30], GB5009.4-2016 [31] and GB5009.9-2023 [32], respectively. Total lipid was determined by lipids analyzer (SZF, Shanghai, China). The composition of lipids was detected by LC-MS (7890A, Agilent, Santa Clara, CA, USA). Amino acids analyzed by automatic amino acid analyzer (LA8080, Hitachi, Tokyo, Japan); fatty acids by GC-MS (7890A, Agilent, USA); flavonoids by HPLC (Agilent 1260, USA) following extraction with methanol and standards calibration.

Liver, muscle and blood tissues of *C. idella* fed with F75D25 diet were added to PBS at pH 7.30–7.50 and then ground into a homogenate. The homogenate was transferred to a centrifuge tube and subjected to centrifugation at 3500 rpm for 6 min. Following centrifugation, the supernatant was carefully aspirated. Then the contents of superoxide dismutase (SOD), glutamate oxaloacetate transaminase (GOT), C-reactive protein (CRP) and lysozyme (LYS) in *C. idella* tissues were determined by a kit provided by ELISA detection system (Jingmei, Biotech, Yancheng, Jiangsu Province, Suzhou, China). Microplate reader provided by public service platform for large-scale instruments and equipment of Guangdong Academy of Sciences.

### 2.5. RNA Extraction, Transcriptomic Analysis, and qRT-PCR Analysis

Sample livers and muscle were collected at three months from *C. idella* fed with the F75D25 diet and the control diet (F100D0) for RNA extraction. Three biological replicates per group (*n* = 3) were used for RNA sequencing. Samples were processed individually, and biological replicates were not pooled prior to DNA extraction, library preparation, or sequencing. Total RNA was extracted, and RNA quality was assessed using a 2100 Bioanalyzer (Agilent, Palo Alto, CA, USA) and quantified using an ND-2000 spectrophotometer (NanoDrop Technologies, Wilmington, DE, USA). Sequencing of each sample was performed on the Illumina platform (PE150) at GENE SENOVO (Shanghai, China). qRT-PCR validation was performed on a CFX Connect real-time system using SYBR Green qPCR Master Mix and a Bio-Rad CFX Real-Time PCR system (Bio-Rad, Hercules, CA, USA), with 18S rDNA (MSTRG.10085) as the internal reference gene (Table S1). The primers used for qRT-PCR were designed using Primer 3 software (Table S2). Relative gene expression levels were calculated using the 2^−ΔΔCT^ method [33].

### 2.6. Genomics DNA Extraction of Gut Microbes

For gut microbiota analysis, six fish were randomly selected per treatment (*n* = 6 biological replicates). Intestinal contents from each fish were collected separately, and DNA of the microbial community was extracted using the MagPure Stool DNA KF kit B (Magen, Guangzhou, China) according to the manufacturer’s protocol. The extracted DNA was quantified using a Qubit Fluorometer with the Qubit dsDNA BR Assay kit (Invitrogen, CHI, Carlsbad, CA, USA), and its quality was assessed by running an aliquot on a 1.00% agarose gel [34].

According to Zhu et al. [35], the main steps of library construction are as follows: Sample testing involves assessing the concentration, integrity, and purity of the sample, with concentration determined by fluorescence quantification or enzyme standardization, and integrity and purity measured on 1% agarose gels (150 V, 40 min). Sample fragmentation is performed by taking 1 µg of genomic DNA and disrupting it using a Covaris instrument with ultrasound. Fragment size selection is done by concentrating the fragmented samples around 200–400 bp using beads. End repair, A base addition, and adapter ligation are carried out next. This is followed by PCR amplification, where the ligated products are amplified, and the amplified products are purified and recovered with magnetic beads. The product cyclization step involves denaturing the PCR products to single strands, then cyclizing them to form single-stranded cyclic products, with uncyclized linear DNA molecules digested to obtain the final library. Library quality control is performed by testing the concentration of the cyclized products before sequencing. Finally, sequencing involves replicating single-stranded circular DNA molecules to form DNA nanoballs (DNBs), which are added to chip mesh pores using high-density DNA nanochip technology and sequenced by co-probe anchored polymerization (cPAS).

### 2.7. Metagenomic Data Analysis

All raw data were trimmed using SOAPnuke v.1.5.2 [36]. High-quality reads were de novo assembled with Megahit software (Version: 1.2.9), and contigs shorter than 300 bp were discarded. Gene prediction on contigs was performed using MetaGeneMarker (2.10), and redundant genes were removed with CD-HIT at a 95% identity cutoff [37]. Taxonomic information was generated by aligning protein sequences of genes against the NR database using DIAMOND with an E-value cutoff of 1 × 10^−5^ and assigning taxonomy based on the MEGAN LCA algorithm [38]. Functional information was obtained by aligning protein sequences against the eggNOG, CAZy, COG, Swiss-Prot, KEGG, and CARD databases using DIAMOND with the same E-value cutoff [39]. Taxonomic and functional abundance profiles were generated by aligning reads to genes using Bowtie 2 with default settings [40]. Features with significantly different abundances across groups were determined using Wilcoxon’s rank sum test, with *p*-values corrected by the BH method (corrected *p*-values < 0.05 considered significant) [41]. Differentially enriched KEGG pathways were identified by reporter scores, with a significance threshold of an absolute reporter score of 1.65 or higher [42]. Alpha diversity was quantified by the Shannon index using relative abundance profiles at gene, genus, and KO levels with the R package (version 2.6.2), while beta diversity was calculated using Bray-Curtis or Jensen-Shannon Divergence distances [43].

### 2.8. Analysis of Experimental Data

All experimental data are reported as means ± standard deviation (S.D.) and were subjected to one-way ANOVA independent-sample f-test and Duncan multiple comparisons using the SPSS for Windows program (version 16.0, CHI, Honolulu, HI, USA). Unless otherwise stated, statistical significance was determined at a level of *p* < 0.05.

All experimental procedures involving animals were approved by the Animal Care and Use Committee of the Guangdong Academy of Agricultural Sciences (Approval No. GAAS2024-AQ-012) and followed relevant national guidelines for the care and use of experimental animals.

## 3. Results and Discussions

### 3.1. Overview of Nutritional Quality of Commercial Feed and S. polyrhiza

As a first step, we analyzed the commercial feed and *S. polyrhiza* meal for proximate composition (protein, lipids, starch, and ash); the results are reported in Table 1. In addition, Table 1 provides the amino-acid profile of proteins, the fatty-acid distribution of total lipids, and the flavonoid compounds for both the commercial feed and *S. polyrhiza* meal. For reference, the ingredient composition of the five experimental diets is shown in Table 2. The protein content of *S. polyrhiza* was 35.43 g/100 g, which was higher than that in commercial feed (28.00 g/100 g). In addition, total lipids content in *S. polyrhiza* (5.87 g/100 g) was also higher than commercial feed with 4.45 g/100 g content. Conversely, the starch and ash content was substantially lower in *S. polyrhiza* (14.50 g/100 g, 7.74 g/100 g) than in commercial feed (30.48 g/100 g, 9.56 g/100 g). The higher levels of lysine, leucine, and glutamic acid in duckweed are particularly noteworthy. Lysine is a limiting amino acid in many plant-based feeds, and its abundance in *S. polyrhiza* can significantly improve the nutritional value of feed formulations [44]. Moreover, the increased levels of amino acids such as serine, isoleucine, arginine, and alanine in duckweed suggest enhanced metabolic and growth benefits [45]. Arginine, for instance, is essential for protein synthesis and the urea cycle, while isoleucine plays a pivotal role in muscle metabolism and immune function [46].

*S. polyrhiza* demonstrates a superior amino acid profile relative to commercial feed. Notable differences include lysine (2.13 g/100 g in *S. polyrhiza* vs. 1.82 g/100 g in f commercial feed), leucine (2.68 g/100 g in *S. polyrhiza* vs. 2.05 g/100 g in commercial feed), and glutamic acid (6.68 g/100 g in duckweed vs. 4.08 g/100 g in commercial feed). *S. polyrhiza* also contains higher levels of serine (1.89 g/100 g), isoleucine (1.78 g/100 g), arginine (2.35 g/100 g), and alanine (2.94 g/100 g). Despite these overall higher values, methionine is an exception, being lower in *S. polyrhiza* (0.46 g/100 g) compared to commercial feed (0.60 g/100 g). This high protein content, coupled with the enhanced profile of essential amino acids, positions duckweed as an excellent source of high-quality protein. The higher levels of lysine, leucine, and glutamic acid in duckweed are particularly noteworthy. Lysine is a limiting amino acid in many plant-based feeds, and its abundance in duckweed can significantly improve the nutritional value of feed formulations.

The fatty acid composition of *S. polyrhiza* is distinctively different from that of commercial feed. Saturated fatty acids (SFA) are lower in *S. polyrhiza* (1.44 g/100 g) compared to commercial feed (2.06 g/100 g). Monounsaturated fatty acids (MUFA) are also slightly lower in *S. polyrhiza* (1.08 g/100 g) compared to commercial feed (1.51 g/100 g). However, polyunsaturated fatty acids (PUFA) are notably higher in *S. polyrhiza* (3.48 g/100 g) than in commercial feed (1.53 g/100 g). Specifically, *S. polyrhiza* is particularly richer in omega-3 fatty acids (2.88 g/100 g) and α-linolenic acid (2.64 g/100 g) compared to commercial feed (1.47 g/100 g and 0.35 g/100 g, respectively). *S. polyrhiza*’s fatty acid profile demonstrates a distinct advantage in terms of polyunsaturated fatty acids (PUFAs). PUFAs, particularly omega-3 fatty acids, are known for their anti-inflammatory properties and benefits to cardiovascular health in animals. These fatty acids are also crucial for improving the quality of animal products, such as meat and eggs, by enhancing their nutritional profile [47]. The reduced levels of saturated fatty acids (SFA) and monounsaturated fatty acids (MUFA) in *S. polyrhiza* further support its use as a healthier feed alternative. The balance of omega-3 to omega-6 fatty acids in duckweed is more favorable (2.88 g/100 g vs. 0.60 g/100 g) compared to conventional commercial feed (1.47 g/100 g vs. 0.81 g/100 g), which is beneficial for reducing inflammation and promoting better health outcomes in animals [48].

Additionally, *S. polyrhiza* is rich in flavonoid compounds, which are not present in measurable amounts in commercial feed. The total flavonoid content in *S. polyrhiza* is 158.43 mg/100 g, with specific compounds including quercetin (28.50 mg/100 g), rutin (24.38 mg/100 g), chlorogenic acid (13.25 mg/100 g), catechin (31.23 mg/100 g), apigenin (10.45 mg/100 g), kaempferol (14.74 mg/100 g), and fisetin (5.65 mg/100 g). These bioactive compounds, including quercetin, rutin, chlorogenic acid, catechin, apigenin, kaempferol, and fisetin, are known for their antioxidant properties. The total flavonoid content in duckweed (158.43 mg/100 g) can confer various health benefits, such as enhanced immune function, reduced oxidative stress, and improved gut health in livestock [49,50,51].

### 3.2. Growth Performance and Muscle Quality of C. idella with Different S. polyrhiza Proportion

**Figure 1:**
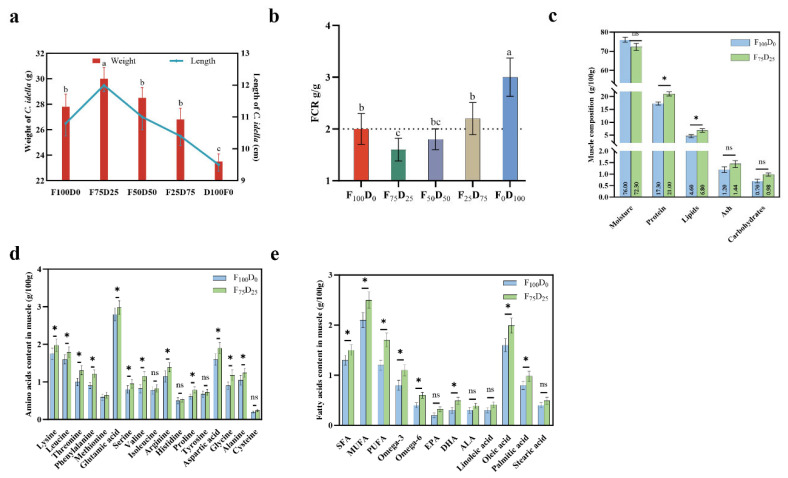
The growth performance and muscle quality of *C. idella* with different proportions of *S. polyrhiza* in the diet. Effect of dietary *S. polyrhiza* inclusion on body weight and length (**a**), feed conversion ratio (FCR) (**b**), muscle composition (**c**), amino acid profile in muscle (**d**), and fatty acid profile in muscle (**e**). * *p* < 0.05, ns: non-significant. Different lowercase letters (a, b, c) indicate statistically significant differences among groups (*p* < 0.05), as determined by one-way ANOVA followed by post hoc multiple comparison tests.

The weight and length measurements of *C. idella* under different diet combinations (F100D0, F75D25, F50D50, F25D75, and D100F0) show significant variations (Figure 1a). The diet with 75% commercial feed and 25% *S. polyrhiza* (F75D25) resulted in the highest weight (30.00 g) and length (12.00 cm), indicating an optimal balance between conventional feed and duckweed for promoting growth. Diets with higher proportions of *S. polyrhiza* (F25D75 and F0D100) led to decreased growth, suggesting that a balanced inclusion of duckweed enhances growth performance more effectively than its exclusive use.

The FCR values, representing the efficiency of feed utilization, vary across the dietary groups (Figure 1b). The F75D25 diet exhibits the lowest FCR (1.60), indicating the most efficient feed conversion compared to other groups. Higher FCR values observed in diets with higher *S. polyrhiza* content (F25D75 and F0D100) suggest less efficient feed utilization, possibly due to the lower digestibility or nutrient availability in duckweed when used alone.

The muscle composition analysis reveals significant differences between the F100D0 and F75D25 groups (Figure 1c). The F75D25 diet results in higher protein (21.00 g/100 g) and lipid (6.50 g/100 g) contents in muscle compared to the F100D0 diet (17.30 g/100 g protein and 4.60 g/100 g lipids). This indicates that the inclusion of duckweed enhances the nutritional quality of muscle tissue. However, moisture, ash and carbohydrates contents remain relatively stable across the diets. Amino acid profiling of muscle tissues demonstrates that the F75D25 diet significantly increases the content of essential amino acids such as lysine, leucine, and threonine compared to the F100D0 diet (Figure 1d). The overall higher amino acid content in the F75D25 group underscores the potential of duckweed to improve the amino acid profile of fish muscle, thereby enhancing its nutritional value. The fatty acid analysis shows significant differences between the F100D0 and F75D25 diets (Figure 1e). The F75D25 diet results in higher levels of polyunsaturated fatty acids (PUFA) and omega-3 fatty acids, including EPA and DHA, compared to the F100D0 diet. This is consistent with the fatty acid profile of duckweed, which is rich in PUFAs and omega-3. The increased omega-3 content, particularly ALA (alpha-linolenic acid), suggests that duckweed can enhance the health benefits of fish muscle by improving its fatty acid profile.

The graded inclusion levels of duckweed meal (0–100%) were selected to provide a stepwise continuum from partial to complete inclusion, allowing us to delineate both the feasibility limits of substitution and the optimal range for grass carp. Within this framework, moderate inclusion (25–50%) maintained or improved crude protein and lipid contents in muscle relative to the control, and enhanced essential amino acids (e.g., lysine, leucine, threonine; Figure 1d) together with higher polyunsaturated and omega-3 fatty acids in muscle (Figure 1e). In contrast, at higher inclusion levels (≥75%), growth performance and feed efficiency declined (Figure 1a,b), suggesting constraints in nutrient availability and potential shifts in lipid deposition and fatty acid balance when duckweed becomes the predominant or sole ingredient. Collectively, these observations indicate that the inclusion level per se is a driver of muscle compositional outcomes, with ~25% inclusion providing the most favorable balance between growth efficiency and flesh quality in this study.

### 3.3. Biochemical Parameters in the Liver, Muscle, and Blood of C. idella

**Figure 2:**
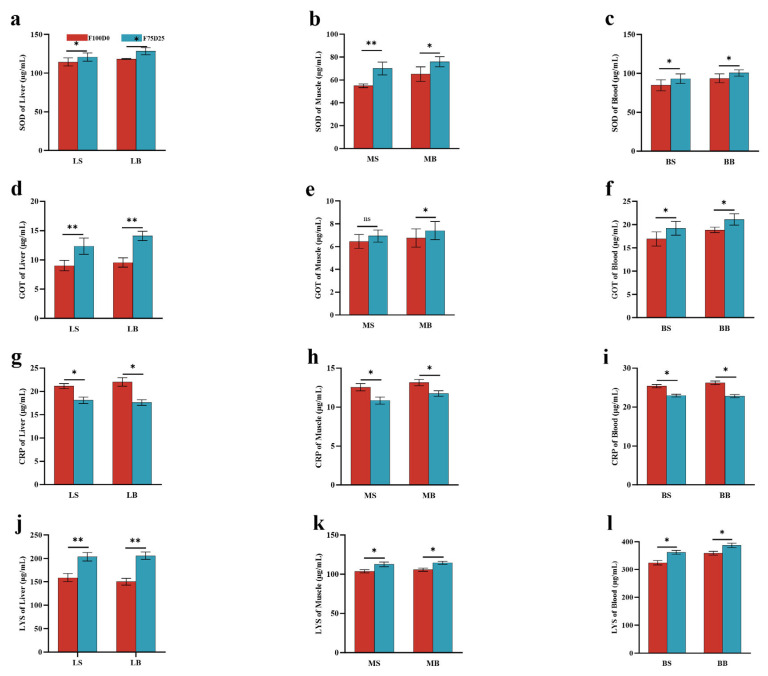
The biochemical parameters in the liver, muscle, and blood of *C. idella* with fed with 25% *S. polyrhiza* (F75D25) and the control diet (F100D0). (**a**–**c**) shown superoxide dismutase (SOD) activity; (**d**–**f**) shown glutamate oxaloacetate transaminase (GOT) levels; (**g**–**i**) shown C-reactive protein (CRP) levels; (**j**–**l**) shown lysozyme (LYS) activity. (LS: one-month grass carp liver; LB: three-month grass carp liver; MS: one-month grass carp muscle; MB: three-month grass carp muscle; BS: one-month grass carp blood; BB: three-month grass carp blood). * *p* < 0.05, ** *p* < 0.01, ns: non-significant.

This study demonstrated that incorporating *S. polyrhiza* at 25% of the diet (F75D25) significantly enhanced various biochemical parameters in fish liver, muscle, and blood compared to a 100% conventional feed diet (F100D0) (Figure 2). The F75D25 group exhibited higher superoxide dismutase (SOD) activity across all tissues, with notable increases in the liver (both at one and three months), muscle, and blood. This is consistent with findings from other studies that have shown the benefits of antioxidant-rich diets in improving fish health and resilience [52]. Glutamate oxaloacetate transaminase (GOT) levels were also elevated in the F75D25 group, particularly in the liver, suggesting enhanced liver function and metabolic activity. This improvement could be attributed to the nutrient-rich composition of duckweed, which provides essential amino acids and other nutrients that support metabolic processes [53]. Additionally, C-reactive protein (CRP) levels were higher in the F75D25 group, indicating a mild inflammatory or adaptive immune response. Lysozyme (LYS) activity was consistently higher in the F75D25 group across all tissues, reflecting enhanced immune function [54]. The study demonstrates that a 25% inclusion of *Spirodela polyrhiza* in the diet of *C. idella* significantly enhances antioxidant, metabolic, and immune parameters in fish liver, muscle, and blood. These findings support the potential of duckweed as a viable and sustainable feed alternative in aquaculture, promoting better fish health and growth performance.

### 3.4. Transcriptomic Analysis of C. idella Reveals Enhanced Immune Response, Muscle Quality, and Amino Acid Composition with F75D25 Diet

#### 3.4.1. Immune-Related Genes in the Liver

**Figure 3:**
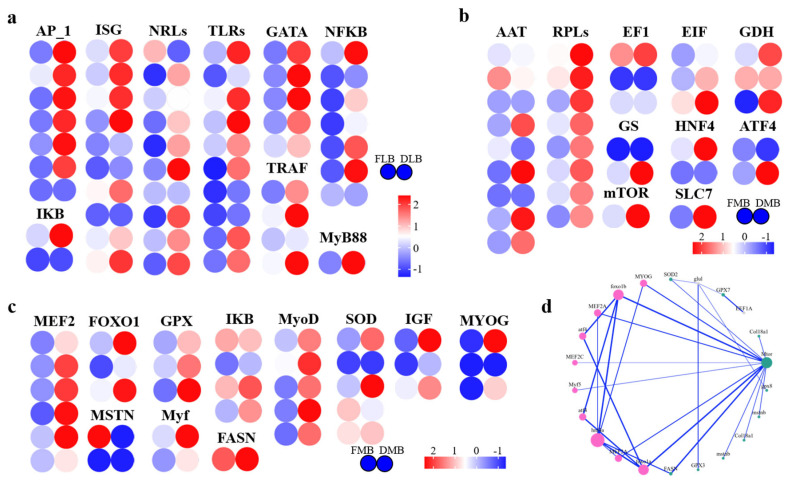
A transcriptomic analysis of *C. idella* fed with F75D25 diet. Figure illustrates the transcriptomic analysis of grass carp liver tissue showing upregulated immune-related genes (**a**), genes related to muscle quality (**b**), genes associated with amino acid composition in muscle (**c**), and the gene correlation network (**d**). (FLB: grass carp liver fed with F100D0; DLB: grass carp liver fed with F75D25. FMB: grass carp muscle fed with F100D0; DMB: grass carp muscle fed with F75D25).

The transcriptomic analysis of the liver tissue revealed significant modulation of genes associated with immune response in grass carp fed with F75D25 diet (DLB) compared to the control group (FLB). Notably, there was an upregulation of several Toll-like receptors (TLRs), including TLR2, TLR3, TLR4, TLR5, and TLR7, which are critical for recognizing pathogen-associated molecular patterns (PAMPs). Additionally, NOD-like receptors (NLRs) such as NOD1 and NOD2, which play roles in recognizing intracellular pathogens, were also upregulated (Figure 3a).

Key signal transduction genes exhibited differential expression. MyD88, a crucial adaptor in TLR signaling pathways, and TRIF, another adaptor mediating antiviral responses through TLR3 and TLR4, showed increased expression. Furthermore, IL-1 receptor-associated kinases (IRAK1 and IRAK4) and TNF receptor-associated factor 6 (TRAF6) were significantly upregulated, suggesting enhanced activation of TLR and IL-1 signaling pathways. Genes involved in inflammatory responses, such as NF-κB, IKKα, and IKKβ, were upregulated, indicating increased activation of inflammatory pathways. Cytokines including TNF-α, IL-1β, and IL-6 also showed elevated expression, highlighting a robust immune response. Genes related to antiviral and antibacterial defenses were notably expressed. Interferons (IFN-α and IFN-β) and interferon-stimulated genes (ISGs) such as Mx, PKR, and OAS were upregulated, enhancing antiviral mechanisms. Additionally, antimicrobial peptides, including chemokines (CCLs) and defensins, were significantly expressed, indicating a strong antibacterial defense. Other key transcription factors, such as STAT1, STAT3, STAT5, AP-1, and GATA3, showed elevated expression levels, further supporting enhanced immune regulation and response. It should be noted that the increased activation of inflammatory pathways may also represent a potential negative effect, as it could indicate the occurrence of inflammatory responses rather than solely beneficial immune stimulation. During the experimental period, no obvious abnormalities in muscle appearance or coloration were observed during routine sampling; however, muscle coloration was not quantitatively assessed and should be examined in future studies.

#### 3.4.2. Genes Related to Muscle Quality in *C. idella*

The muscle tissue transcriptomic analysis identified several genes related to muscle growth and quality (Figure 3b). Myostatin (MSTN), a negative regulator of muscle growth, was downregulated, suggesting potential increases in muscle mass. Conversely, genes promoting muscle differentiation and growth, such as Myogenin (MYOG), Myogenic factor 5 (Myf5), and Myogenic differentiation 1 (MyoD), were upregulated. Additionally, Insulin-like growth factor 1 (IGF-1), known to enhance muscle growth and regeneration, showed increased expression.

Genes involved in fat metabolism, including PPARα and PPARγ, were differentially expressed, indicating altered fatty acid oxidation and storage. Fatty acid-binding proteins (FABP3) and fatty acid synthase (FASN), key in fatty acid transport and synthesis, showed significant expression changes. SREBP-1c, a transcription factor regulating lipid synthesis, was also upregulated. Collagen synthesis genes, such as COL1A1 and COL1A2, were differentially expressed, potentially affecting meat texture and tenderness. Antioxidant genes, including SOD1 and GPX1, were upregulated, suggesting enhanced protection against oxidative stress in muscle tissue. Key transcription factors involved in muscle growth and quality, such as MyoG, FOXO1, and MEF2, exhibited significant expression changes, supporting improved muscle development and regeneration.

#### 3.4.3. Genes Related to Amino Acid Composition in Muscle

The analysis of muscle tissue revealed significant modulation of genes associated with amino acid metabolism (Figure 3c). Elongation factor 1-alpha (EF1α) and ribosomal proteins (RPL3 and RPL5), essential for protein synthesis, were upregulated. The mechanistic target of rapamycin (mTOR), a crucial regulator of protein synthesis and cell growth, showed increased expression.

Amino acid metabolism genes, such as amino acid transporters (AATs) including SLC1A5 (ASCT2), were upregulated, indicating enhanced amino acid transport. Enzymes involved in amino acid synthesis and metabolism, such as glutamine synthetase (GS) and glutamate oxaloacetate transaminase (GOT), were differentially expressed. Glutamate dehydrogenase (GDH) also showed significant changes, suggesting alterations in amino acid conversion processes. Genes responsible for amino acid transport, such as SLC7A5 and SLC38A2, exhibited increased expression, supporting enhanced nutrient uptake and utilization. Transcription factors regulating protein metabolism, including ATF4, c-Myc, and HNF4α, were significantly expressed, promoting amino acid metabolism and protein synthesis.

Signaling pathways critical for protein synthesis, such as the PI3K/AKT/mTOR pathway and AMPK, showed differential expression, indicating enhanced regulation of cell growth and metabolism. Other important genes and transcription factors, including EIF2 and FOXO, were upregulated, further supporting improved protein synthesis and amino acid metabolism.

Additionally, the gene correlation network analysis (Figure 3d) illustrated the interactions between immune response, muscle quality, and amino acid composition genes. This network highlights the complex regulatory mechanisms and interplay among these biological processes, underpinning the multifaceted benefits of duckweed inclusion (F75D25) in grass carp.

The results demonstrate that dietary inclusion with 25% duckweed significantly enhances immune responses, improves muscle quality, and optimizes amino acid composition in grass carp. The upregulation of key immune-related genes may reflect enhanced immune responsiveness or immune priming; however, such transcriptional changes can also occur under inflammatory or stress-related conditions and do not necessarily indicate improved disease resistance. Although no clinical signs of disease or abnormal mortality were observed during the experiment, these responses should be interpreted cautiously and warrant further validation through pathogen challenge tests in future studies [55,56]. Enhanced expression of muscle growth and quality genes indicates potential improvements in muscle mass and texture, consistent with findings on the modulation of myostatin and myogenic factors [57]. Furthermore, the upregulation of amino acid metabolism genes supports improved nutrient utilization and protein synthesis, contributing to overall better growth performance and health [58,59,60]. These findings provide valuable insights into the molecular mechanisms underlying the beneficial effects of duckweed inclusion, promoting sustainable and efficient fish farming practices.

### 3.5. Gut Microbiota Composition and Enhances Functional Capabilities in C. idella with F75D25

**Figure 4:**
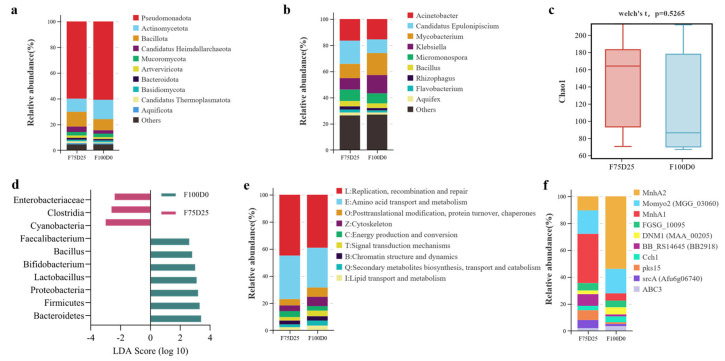
The gut microbiota composition and functional capabilities in C. idella fed with F75D25 diet. The figure shows the gut microbiota composition at phylum (a) and genus levels (b), alpha diversity analysis (c), LEfSe analysis (d), eggNOG functional analysis (e), and PHI functional analysis (f) in grass carp fed with 25% *S. polyrhiza* (F75D25) and control diet (F100D0).

The gut microbiota composition of grass carp fed with diets where 25% of the commercial feed was replaced with duckweed (F75D25) and a control diet without duckweed (F100D0) was analyzed at both phylum and genus levels (Figure 4a,b). At the phylum level, Pseudomonadota dominated in both groups, accounting for over 80% of the total bacterial population. Other phyla, such as Actinomycetota, Bacillota, and Bacteroidota, were present in relatively lower abundances (Figure 4a). The presence of Pseudomonadota as the dominant phylum is consistent with previous studies indicating its significant role in the gut microbiota of freshwater fish [61]. At the genus level, the most abundant genera included *Acinetobacter*, *Candidatus Epelionipiscium*, and *Mycobacterium*, with varying proportions between the two groups (Figure 4b). The relative abundance of *Acinetobacter* was higher in the F75D25 group compared to the F100D0 group. *Acinetobacter* is known for its beneficial role in nutrient cycling and degradation of complex organic materials, which could be advantageous for fish health and growth [62,63].

Alpha diversity analysis, represented by the Chao1 index, was conducted to assess the species richness within each group (Figure 4c). The results indicated no significant difference in species richness between the F75D25 and F100D0 groups (*p* = 0.5265). This suggests that duckweed inclusion does not significantly alter the overall diversity of the gut microbiota. Maintaining a stable microbiota diversity is crucial for gut health and function, as high diversity is often associated with resilience against pathogens and improved metabolic capabilities [64].

The linear discriminant analysis effect size (LEfSe) analysis identified several bacterial taxa that were differentially abundant between the two groups (Figure 4d). Notably, the relative abundance of *Bacillus* was higher in the F75D25 group. *Bacillus* species are well-known probiotics that enhance gut health, improve digestion, and provide immunomodulatory effects [65]. This increase suggests that duckweed inclusion may contribute to a more beneficial gut microbiota composition.

Functional profiling using eggNOG revealed significant differences in several functional categories between the two groups (Figure 4e). Genes related to replication, recombination, and repair were more abundant in the F75D25 group, indicating enhanced cellular repair mechanisms. Amino acid transport and metabolism, as well as posttranslational modification, protein turnover, and chaperones, were also more prevalent in the duckweed-supplemented group. These functional enhancements could be linked to improved nutrient absorption and protein synthesis, crucial for the growth and health of grass carp [66]. Pathogen-Host Interaction (PHI) analysis showed variations in genes related to bacterial virulence factors between the groups (Figure 4f). The F75D25 group exhibited higher levels of genes associated with beneficial traits, such as stress response (e.g., MnhA1, MnhA2) and reduced expression of genes linked to pathogenicity (e.g., pks15). This suggests that duckweed inclusion may not only support beneficial gut bacteria but also reduce the potential pathogenic load, contributing to overall better fish health.

The present study demonstrates that dietary inclusion of 25% duckweed significantly influences the gut microbiota composition and functional capabilities of grass carp. The dominance of *Pseudomonadota* and the increased relative abundance of beneficial genera such as *Acinetobacter* and *Bacillus* suggest that duckweed can modulate the gut microbiota in a way that enhances nutrient cycling and gut health. The alpha diversity analysis indicated stable microbiota diversity between the two groups, highlighting that duckweed inclusion does not disrupt the overall microbial community structure. This stability is essential for maintaining gut health and preventing dysbiosis [64].

Functional analyses revealed that duckweed inclusion enhances genes related to cellular repair, amino acid transport, and metabolism, as well as protein turnover. These functional improvements can lead to better nutrient utilization and overall fish growth. Moreover, the reduction in pathogenicity-related genes suggests a potential protective effect against gut pathogens, which could reduce disease incidence and improve fish welfare [66].

Overall, these findings align with previous research on the benefits of plant-based feed additives in aquaculture, emphasizing the potential of duckweed as a sustainable and health-promoting dietary component for grass carp [67,68]. Future studies should explore the long-term effects of duckweed inclusion on fish health and productivity, as well as its potential to replace conventional feed ingredients in aquaculture diets.

It should be noted that duckweed species are known to effectively accumulate heavy metals such as cadmium, lead, copper, and zinc from aquatic environments. Although the duckweed used in this study was sourced from a clean freshwater culture system, the concentrations of heavy metals in the duckweed biomass were not directly quantified. This represents a limitation of the present study. Elevated heavy metal levels in duckweed could potentially influence physiological, biochemical, and immune-related responses in fish. Therefore, future studies should include systematic monitoring of heavy metal contents in duckweed-based feeds to fully evaluate their safety and long-term effects on fish health.

## 4. Conclusions

This study demonstrated that the graded inclusion of commercial feed with *S*. *polyrhiza* meal had species-specific effects on grass carp growth, nutrient utilization, muscle quality, immune responses, and gut microbial communities. While duckweed has previously been tested as an aquafeed ingredient, our findings extend this knowledge by systematically evaluating a full gradient of inclusion levels (0–100%) under controlled conditions and linking dietary treatments with multi-level responses from growth performance to transcriptomics and metagenomics. The results indicate that moderate inclusion (around 25–50%) can sustain growth while improving flesh quality and enhancing the profile of beneficial fatty acids, whereas excessive inclusion (≥75%) may compromise performance. These outcomes highlight the dual value of duckweed as both a sustainable protein source and a functional dietary component capable of influencing host metabolism and microbiota. Importantly, this work provides an evidence-based reference for defining optimal inclusion thresholds in grass carp diets and contributes to ongoing efforts toward more sustainable aquaculture production.

## Figures and Tables

**Table 1:** Nutritional composition of commercial feed and *S. polyrhiza.*

Category of Nutrients	Nutrient	Content (g/100 g Dry Weight)	
		Commercial Feed	*S. polyrhiza*
Basic Components	Protein	28.00	35.43
	Starch	30.48	14.50
	Total Lipids	4.45	5.87
	Ash	9.56	7.74
Amino Acid Composition	Lysine	1.82	2.13
	Leucine	2.05	2.68
	Threonine	1.24	1.61
	Phenylalanine	1.31	1.72
	Methionine	0.60	0.46
	Glutamic Acid	4.08	6.68
	Serine	1.25	1.89
	Isoleucine	1.18	1.78
	Arginine	1.67	2.35
	Histidine	0.70	0.74
	Proline	1.12	1.58
	Tyrosine	1.05	1.46
	Glycine	1.53	2.56
	Alanine	2.03	2.94
	Cysteine	0.40	0.49
Fatty Acid Composition	SFA	2.06	1.44
	MUFA	1.51	1.08
	PUFA	1.53	3.48
	Omega-3 Fatty Acids	1.47	2.88
	Omega-6 Fatty Acids	0.81	0.60
	α-Linolenic Acid	0.35	2.64
	Linoleic Acid	0.80	0.60
	Oleic Acid	1.03	0.96
	Palmitic Acid	1.09	1.20
	Stearic Acid	0.55	0.24
Flavonoid Compounds (mg/100 g)	Total Flavonoids	-	158.43
	Quercetin	-	28.50
	Rutin	-	24.38
	Chlorogenic Acid	-	13.25
	Catechin	-	31.23
	Apigenin	-	10.45
	Kaempferol	-	14.74
	Fisetin	-	5.65

Note: The nutrient composition of the commercial feed and *S. polyrhiza* meal was determined by laboratory analysis: protein by the Kjeldahl method; lipids by Soxhlet extraction; amino and fatty acids by LC-MS and GC-MS, respectively; and flavonoid compounds by HPLC using methanol extraction and standard calibration.

**Table 2:** The ingredient formulation (g/kg dry matter; totals = 1000 g/kg) and proximate composition (% DM) of the experimental diets for *C. idella*. Each diet was formulated on a dry-matter basis with Commercial feed (CF) and *S. polyrhiza* meal summing to 1000 g/kg. Values for fish meal, wheat starch, and vegetable oil represent the within-diet contributions originating from the CF fraction (i.e., components already present in the Commercial feed), not extra additions beyond 1000 g/kg. Proximate composition values were obtained by mass-weighted averaging of the Commercial feed and *S. polyrhiza* compositions listed in Table 1 to ensure isonitrogenous and isoenergetic formulations.

Component (g/kg DM)	F100D0	F75D25	F50D50	F25D75	F0D100
Commercial feed	1000.0	720.0	480.0	240.0	0.0
Duckweed meal	0.0	240.00	480.0	720.0	940.0
Added fish meal	0.0	10.0	15.0	5.0	0.0
Added wheat starch	0.0	10.0	15.0	20.0	30.0
Added vegetable oil	0.0	20.0	10.0	15.0	30.0
Total (g/kg DM)	1000.0	1000.0	1000.0	1000.0	1000.0
**Proximate composition (% DM)**					
Crude protein	28.0	28.8	29.5	30.2	30.8
Crude lipid	4.50	4.9	5.2	5.4	5.8
Starch	30.5	28.5	26.5	24.5	22.5
Ash	9.6	9.3	9.1	8.8	8.5

Note: Crude protein, lipid, starch, and ash contents were determined according to GB5009 standards.

## Data Availability

The data that support the findings of this study are availability from the corresponding author upon reasonable request.

## Supplementary Materials

The following supporting information can be downloaded at https://www.mdpi.com/article/10.3390/ani16010053/s1, Table S1. The Gene sequence of internal standard for qRT-PCR analysis. Table S2. The Primer sequence of DEGs for qRT-PCR analysis. Figure S1. Relative expression levels of key genes in grass carp measured by qRT-PCR.
