# Supplementary material for: Duckweed as a Sustainable Aquafeed: Effects on Growth, Muscle Composition, Antioxidant and Immune Markers in Grass Carp
Source: Animals (Basel). 2025 Dec 24;16(1):53. doi: 10.3390/ani16010053 (PMC12784702; doi:10.3390/ani16010053)
Supplement: Supplementary file 1 [file animals-16-00053-s001.zip › animals-3852445-supplementary.pdf]

Table S1 The Gene sequence of internal standard for qRT-PCR analysis

Gene ID	ce
MSTRG.10085	TTGTGGTAATTAGAAGCCAAATGGGTCTTGTAAATTCTTTAAGATGCTACAAGG<br>A<br>AGAGGTCAATGGAGGATAGGTGTTGGGCGGTTTGGGGGAAGACTGCATTATGA<br>GT<br>CTGGGTGGAAAACTGCACTATAATTTCTCATTTTCCTTCTTTTGTTTGCCTGTTC<br>AGGTCTCAGCATTTTTGGACAGCATATTGTACTGAAGACTTGAAAGAATGTTAG<br>A<br>TCTTTCCTTGTATGACCAAAGTCTTCTGGCCAGCCCATCCAAGGATCCAAGACC<br>A<br>CAGGCCTCACAACCATCAGACTCTGCTGTGGCTTTCTGAACCCATTGCTGCACC<br>T<br>CTTT

Table S2 The Primer sequence of DEGs for qRT-PCR analysis

Gene Name	ces of primers (5' to 3')
AP_1-F	TCCGCTCTCGAGTCT
AP_1-R	GTGGCCTTCTCAGAA
NRLs-F	GCTGTACGCTCGCCC
NRLs-R	TCGCTCTCCTTGACG
GATA-F	GTCCAGGAGGAGCGC
GATA-R	CCCTGCTCGACCTGGT
RPLs-F	TGCTTCGTGGCCGTA
RPLs-R	ACATTGTGCTCAGCG
SLC7-F	GCGTTGAGTTCGAGG
SLC7-R	ATCTGCATCAAAGGC
MSTN-F	GATGCCGTTGAGCGT
MSTN-R	TTGGCGGTATCCATG
SOD-F	CACCACGTGCACCAT
SOD-R	CCCCGGAAGGACTTA

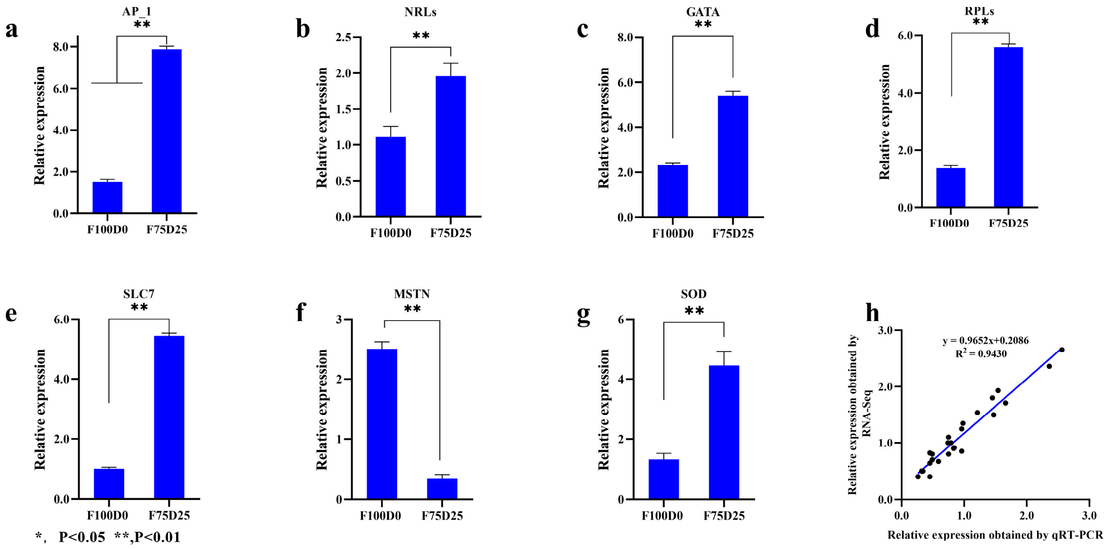

Figure S1 qRT-PCR validation of differentially expressed genes.

(a–g) Relative expression levels of AP-1, NRLs, GATA, RPLs, SLC7, MSTN, and SOD in the F100D0 and F75D25 groups, determined by qRT-PCR. Gene expression levels were normalized to the internal reference gene and calculated using the $2^{-\Delta\Delta Ct}$ method. Data are presented as mean ± SD (n = 3). Statistical significance between groups was analyzed using Student's t-test. (h) Correlation analysis between gene expression levels obtained by RNA-seq and qRT-PCR, showing strong consistency between the two methods ($R^2 = 0.9430$). $P < 0.01$ (**).
